# Comparing Merocel versus Surgicel as nasal packing for post-inferior turbinoplasty surgery

**DOI:** 10.1017/S0022215126104733

**Published:** 2026-06

**Authors:** Christina H. Ng, Elaine Huang, Khadijah Azman, David Y.M. Low, James W.M. Kwek, Maria C.Y. Pang, Ian Chi Yuan Loh, Tee Sin Lee

**Affiliations:** 1 Department of Otolaryngology/Head & Neck Surgery, Changi General Hospital, Singapore, Singapore; 2 Department of Otolaryngology/Head & Neck Surgery, Sengkang General Hospital, Singapore, Singapore; 3 National University of Singapore, Singapore, Singapore; 4 National Technological University, Singapore, Singapore

**Keywords:** haemostasis, haemostatics, nasal obstruction, Merocel, nasal surgical procedures, pain, post-operative complications, quality of life, Surgicel

## Abstract

**Objectives:**

This study aimed to compare Merocel and Surgicel nasal packing following inferior turbinoplasty, focusing on post-operative bleeding, pain, discomfort and nasal obstruction.

**Methods:**

A randomised controlled trial (2017–2021) was conducted in the Department of Otolaryngology, Changi General Hospital, Singapore. Sixty adults undergoing inferior turbinoplasty and/or septoplasty were randomised to receive Merocel or Surgicel packing. Standardised surgical and post-operative protocols were used. Outcomes—bleeding, pain, discomfort and nasal obstruction—were assessed on post-operative day 1 and post-operative days 5–7 using validated scales.

**Results:**

Fifty-eight patients completed the study (Merocel = 30; Surgicel = 28). On post-operative day 1, Surgicel had significantly lower nasal obstruction scores (1.57 ± 0.74 vs 2.10 ± 0.71; *p* = 0.008). By post-operative days 5–7, Merocel showed significantly less bleeding (0.77 ± 0.63 vs 1.18 ± 0.86; *p* = 0.044). Pain and discomfort were comparable.

**Conclusion:**

Merocel provided superior sustained haemostasis, while Surgicel offered better early comfort. Both materials have comparable outcomes.

## Introduction

Nasal obstruction is a cardinal symptom of allergic rhinitis and is frequently refractory to medical therapy when structural causes are present.^1^^–^^3^ It is also recognised as the most burdensome symptom, significantly impairing quality of life.^4^ Amongst structural causes, inferior turbinate hypertrophy is well established as a major contributor to nasal obstruction in allergic rhinitis, often warranting surgical intervention to alleviate fixed-obstruction and aid in dynamic compliance of the nasal airway.

Inferior turbinoplasty is a commonly performed surgical procedure aimed at reducing hypertrophied turbinates.^5^ One of the most notable complications of this surgery is post-operative bleeding,^6^ which can be both troublesome and distressing for patients. Intra-operative haemostasis is typically achieved using electrocauterisation, while post-operative control may be attained with the use of nasal packing.

Various nasal packing options include (but are not limited to) Merocel (Medtronic Inc., Minneapolis, MN, USA),^7^^,^^8^ Surgicel (Ethicon, Somerville, NJ), Nasopore (Polyganics, Groningen, The Netherlands)^8^ and Rapid Rhino (Arthrocare, Knaresborough, UK)^7^^,^^9^ packs. In Singapore, Merocel and Surgicel are amongst the most frequently used options. Merocel is a compressed, dehydrated sponge composed of hydroxylated polyvinyl acetate which exhibits expansion in size upon hydration with normal saline, providing a mechanical tamponade effect. In contrast, Surgicel is a haemostatic agent made of oxidised regenerated cellulose and works by inducing platelet aggregation and formation of a clot matrix. It is bioabsorbable and degrades within days and may often be incorporated into crusts formed over healing turbinate mucosa.

While nasal packing is effective in achieving haemostasis, it is often associated with patient discomfort and impaired breathing while the packing remains in situ. Despite the widespread use of Merocel and Surgicel, no head-to-head clinical studies have compared their efficacy in terms of both haemostasis and patient comfort.

This study aims to address this gap by conducting a comparative analysis of Merocel and Surgicel, evaluating outcomes such as post-operative bleeding, patient-reported pain, nasal obstruction and overall discomfort.

## Methods

This is a prospective, randomised controlled trial conducted between 2017 to 2021 in the Otolaryngology Department of Changi General Hospital, Singapore. We aimed to compare the efficacy of Merocel versus Surgicel for post-operative haemostasis following inferior turbinoplasty. Given that inferior turbinoplasty is often performed in conjunction with septoplasty to address nasal obstruction in patients with allergic rhinitis, patients with concurrent septoplasty performed were also included in our study. Informed consent was attained from patients recruited in the study.

### Inclusion and exclusion criteria

Patients aged 18 years and above with persistent inferior turbinate hypertrophy refractory to optimal medical management and who consented to inferior turbinoplasty (with or without septoplasty) were included.

Exclusion criteria for our study comprised patients with other coexisting nasal conditions such as chronic rhinosinusitis. Patients with prior inferior turbinate reduction surgery were also excluded to avoid confounding from altered vascularity and mucosal integrity. Additionally, patients with congenital or acquired blood disorders, including those receiving oral antiplatelet or anticoagulants, were excluded from this study due to their increased susceptibility to bleeding complications.

### Randomisation

Eligible patients were randomised via a computer-generated sequence into either the Merocel or Surgicel group. Due to the nature of the intervention, blinding of patients and surgeons was not possible. Apart from choice of nasal packing, standardised operative procedures and post-operative care were carried out for both groups.

### Surgical procedure

A training video was shared with participating surgeons (Associate Consultants and above) prior to the commencement of the study. Inferior turbinoplasties were performed in a standardised manner: a medial subperiosteal flap was raised, the turbinate was infractured and excess bone and mucosa were excised. The flap was then replaced, and the remnant turbinate subsequently outfractured. After achieving satisfactory intra-operative haemostasis with diathermy, the surgeon opened a sealed envelope indicating the allocated packing. For patients undergoing concurrent septoplasty, a standardised endoscopic (closed) septoplasty technique was performed. Septal mucoperichondrial flaps were elevated, deviated cartilaginous and/or bony components were corrected as indicated and haemostasis was achieved with diathermy. Bilateral silicone septal splints were inserted in all septoplasty cases and secured to the membranous septum using 2/0 silk sutures. No quilting sutures were utilised. Splints were routinely removed at 1 week post-operatively.

In the Merocel group, an 8 cm Merocel Standard Nasal Dressing packing was lubricated with tetracycline ointment, inserted into bilateral anterior nasal cavities and hydrated with saline to achieve tamponade at the end of surgery. In the Surgicel group, Surgicel was trimmed into standardised sheets and directly applied to raw surfaces of residual inferior turbinates at end of surgery.

### Post-operative care

Post-operative and discharge medications were standardised in both treatment groups. All patients were given oral Paracetamol 500 mg/Codeine phosphate 30 mg three times a day (as necessary) for 1 week and isotonic nasal douche twice daily (BD) for 1 month. All patients were prescribed a 1-week course of oral amoxicillin–clavulanate (Augmentin) 1 g twice daily, in accordance with prevailing institutional practice at the time of the study, as prophylaxis against infection related to inferior turbinate crusting and septal splints in-situ. In our study, non-steroidal anti-inflammatory drugs were withheld to prevent potential exacerbation of bleeding risks.

All patients were admitted for overnight observation and discharged on POD 1. Patients in the Merocel group were surveyed on POD 1 after packing removal, allowing them to reflect on their experience of nasal obstruction while the pack was in situ, as well as any discomfort associated with both its presence and removal. This timing was intended to provide a more comprehensive capture of overall packing experience.

Patients in the Surgicel group were surveyed on POD 1 and discharged with Surgicel left in situ. Outpatient follow-up occurred between POD 5–7, during which nasal toilet and patient interviews were performed. Septal splints were removed when relevant.

### Outcome measures

Primary outcomes measured include patient discomfort, pain, bleeding and nasal obstruction. Discomfort was assessed using visual analog scale (VAS) from 0 (no discomfort) to 10 (worst possible discomfort). Pain was assessed using a pain numerical scale ranging from 0 (no pain) to 10 (worst pain).

In our institution, it is standard post-operative practice to place external nasal bolsters (folded gauze placed beneath the nares) to collect anterior nasal discharge or bleeding that may occur following surgery. Any bleeding that occurred between POD 0 and POD 7 was graded from 0 to 4, with 0 indicating no bleeding; 1 indicating three or less fully-soaked nasal bolster change, 2 indicating more than three fully-soaked nasal bolster changes, 3 indicating bleeding that necessitated cauterisation in clinic and 4 indicating bleeding that required surgical intervention in the operating theatre. Overnight bleeding scores on POD 1 were reported by medical staff, while POD 5–7 bleeding scores were self-reported by patients.

We employed the “Nasal Congestion” domain of the Total Nasal Symptom Score^10^ (TNSS) to ensure uniformity. This was measured with scores from 0 to 3, with 0 indicating no nasal congestion, 1 representing mild congestion (symptoms clearly present but easily tolerated), 2 denoting moderate congestion (symptoms bothersome but tolerable) and 3 indicating severe congestion (symptoms difficult to tolerate, interfering with daily activities).

### Statistical analysis

All statistical analyses were performed with IBM Statistical Package for Social Sciences (SPSS) 13.0 software. The Mann–Whitney *U*-test was employed to assess differences in outcome scores for POD 1 and POD 5–7 between Merocel and Surgicel groups for each outcome measure. Statistical significance was set at *p* less than 0.05.

### Ethics approval

The study received ethical approval from the SingHealth Centralised Institutional Review Board. Upon completion of the first outpatient visit, participants were remunerated with 50 Singapore Dollars (SGD) as a gesture of gratitude for their involvement. The study was funded by Changi General Hospital Research Grant (grant number CHF2016.13-S)

## Results

**Figure 1.**
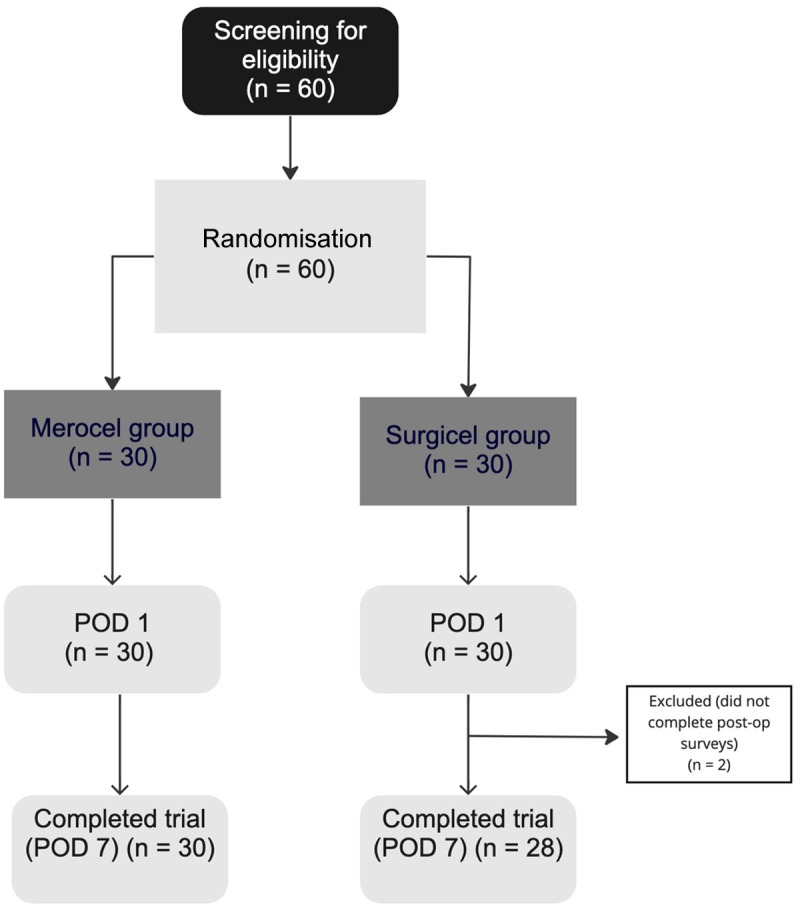
CONSORT diagram showing progression of participants. CONSORT = Consolidated Standards of Reporting Trials; POD = post-operative day.

A total of 60 patients were enrolled for this study and randomly assigned to either Merocel (n=30) or Surgicel (n=30) group. Two patients in the Surgicel group did not complete post-operative follow-up and were consequently excluded from final analysis. As such, the final cohort comprised 58 patients, with 30 in the Merocel group and 28 in the Surgicel group (Figure 1).

### Demographics

The mean age was comparable between the two groups: 29.3 years (SD 10.7) in the Merocel group and 29.8 (SD 11.9) in the Surgicel group. In terms of gender distribution, the Merocel group comprised 22 males (73.3%) and eight females (26.7%), while the Surgicel group comprised 22 males (78.6%) and six females (21.4%). Ethnicity distribution closely reflected that of the Singapore population, with a predominance of Chinese patients in both groups (70.0% [*n* = 21] in Merocel, 85.7% [*n* = 24] in Surgicel). Other ethnic groups included Malays (6.7% [*n* = 2] in Merocel, 0.0% [*n* = 0] in Surgicel), Indians (10.0% [*n* = 3] in Merocel, 3.6% [*n* = 1] in Surgicel) and Others (13.3% [*n* = 4], in Merocel and 10.7% [*n* = 3] in Surgicel) comprised the rest of our study population. Regarding smoking status, the majority of patients were non-smokers (76.7% [*n* = 23] in Merocel, 85.7% [*n* = 21] in Surgicel).

Concurrent septoplasty was performed in 73.3 per cent (*n* = 22) of patients in the Merocel group and 82.1 per cent (*n* = 23) of patients in the Surgicel group. Overall, there was a fairly equal distribution of age, gender, ethnicity, smoking status and concurrent septoplasty in both groups (Table 1).

**Table 1.** Demography Table 1 long description.

	Method of nasal packing	
Parameter	Merocel	Surgicel
	n = 30	n = 28
Age in years		
Mean (SD)	29.3 (10.7)	29.8 (11.9)
Gender (%)		
Male	22 (73.3)	22 (78.6)
Female	8 (26.7)	6 (21.4)
Ethnicity (%)		
Chinese	21 (70.0)	24 (85.7)
Malay	2 (6.7)	0 (0.0)
Indian	3 (10.0)	1 (3.6)
Others	4 (13.3)	3 (10.7)
Smoking status (%)		
Non smoker	23 (76.7)	21 (75.0)
< 5 sticks/day	0 (0.0)	4 (14.3)
5−9 sticks/day	4 (13.3)	1 (3.6)
10−14 sticks/day	2 (6.7)	2 (7.1)
≥ 20 sticks/day	1 (3.3)	0 (0.0)
With septoplasty (%)		
Yes	22 (73.3)	23 (82.1)
No	8 (26.7)	5 (17.9)

SD = standard deviation.

### POD 1 outcomes

At POD 1, patients in the Surgicel group reported significantly lower nasal obstruction scores compared to those in Merocel group (mean ± SD: 1.57 ± 0.74 vs. 2.10 ± 0.71 respectively, *p* = 0.008).

The discomfort level was also lower in the Surgicel group than the Merocel group (3.54 ± 2.35 vs 4.30 ± 2.64, p = 0.249), although this difference was not statistically significant (p=0.249).

Pain scores (2.37 ± 2.17 for Merocel, 2.5 ± 2.22 for Surgicel, p = 0.818) and bleeding scores (1.00 ± 0.53 for Merocel,1.18 ±0.39 for Surgicel, p = 0.146) did not reach statistically significant difference. These findings are summarised in Table 2.

**Table 2.** Comparison of outcome measures on POD 1 vs POD 5–7 between Merocel and Surgicel groups Table 2 long description.

Parameter	Merocel n = 30	Surgicel n = 28	Adjusted *p*-value*
POD 1			
Bleeding			
Mean (SD)			
Range	1 (0.53)	1.18 (0.39)	0.146
Discomfort level	0–2	1–2	
Mean (SD)			
Range	4.3 (2.64)	3.54 (2.35)	0.249
Pain scale	0–10	0–9	
Mean (SD)			
Range	(2.17)	(2.22)	0.818
Obstruction	0–8	0–8	
Mean (SD)			
Range	(0.71)	1.57 (0.74)	**0.008**
	1–3	0–3	
POD 5–7			
Bleeding			
Mean (SD)			
Range	0.77 (0.63)	1.18 (0.86)	**0.044**
Discomfort level	0–3	0–3	
Mean (SD)			
Range	2.63 (2.48)	3.57 (2.3)	0.141
Pain scale	0–7	1–8	
Mean (SD)			
Range	1.80 (2.02)	2.32 (2.21)	0.354
Obstruction	0–7	0–6	
Mean (SD)			
Range	1.20 (0.81)	1.32 (0.72)	0.548
	0–3	0–3	

* Mann–Whitney *U*-test was applied, with significance set at *p* less than 0.05.

POD = post-operative day; SD, standard deviation.

### POD 5–7 outcomes

At the first outpatient follow-up (POD 5–7), patients in the Merocel group demonstrated significantly lower bleeding scores compared to those in Surgicel group (0.77 ± 0.63 vs 1.18 ± 0.86, *p* = 0.044).

Although the discomfort score was numerically lower in the Merocel group (2.63 ± 2.48) than in the Surgicel group (3.57 ± 2.30), the difference was not statistically significant (*p* = 0.141).

Similarly, pain scores (1.80 ± 2.02 for Merocel, 2.32 ± 2.2 for Surgicel, *p* = 0.354) and nasal obstruction scores (1.20 ± 0.81 for Merocel, 1.32 ± 0.72 for Surgicel, *p* = 0.548) were not statistically different. A full comparison of outcome measures between groups at POD 1 and POD 5–7 is detailed in Table 2.

## Discussion

A review of current literature reveals a paucity of recent studies directly comparing Merocel and Surgicel for post-operative nasal packing. A study by Shinkwin *et al*.^11^ investigated the clinical efficacy of Surgicel relative to Vaseline ribbon gauze or Merocel by applying Surgicel to one nostril and randomising the contralateral nostril to either Vaseline gauze or Merocel. Their findings suggested that Surgicel was associated with lower discomfort, both while in situ and during removal, when compared to other packing materials.

In contrast, our study did not demonstrate a significant difference in patient-reported discomfort and pain scores between the Merocel and Surgicel groups. This discrepancy may be attributed to differences in study design. Unlike Shinkwin’s unilateral comparison, our study utilised bilateral placement of the same nasal packing material, thereby eliminating the potential for intra-patient comparison bias. By ensuring patients were unable to discern between packing types across nostrils, our study design sought to minimise reporting bias and improve internal validity.

Our results demonstrated that nasal obstruction on POD 1 was significantly greater in the Merocel group, likely due to its hydrophilic expansion upon hydration, which enhances the tamponade effect but increases obstruction. This effect, however, appeared to be transient, with no significant difference in obstruction noted in POD 5–7. Of interest, despite the initial increase in obstruction, Merocel did not actually result in significantly higher discomfort or pain by POD 1, suggesting that the obstruction may be perceptually tolerable while it was in situ and short-lived as it was removed on POD 1. Although discomfort scores at POD 5–7 were not statistically different between the groups, the Merocel group reported lower absolute scores compared to the Surgicel group (2.63 ± 2.48 vs 3.57 ± 2.30, p = 0.141). This may be attributed to the integration of Surgicel into post-operative nasal crusting, potentially prolonging nasal irritation.

Regarding bleeding control, while no significant difference was observed between the groups on POD 1, Merocel was associated with significantly lower bleeding scores by POD 5–7 (0.77 ± 0.63 in Merocel vs 1.18 ± 0.86 in Surgicel, p=0.044). This finding suggests that a more effective initial tamponade may help to promote longer-term haemostasis. Of the five patients who required cauterisation in clinic by POD 7, four were from the Surgicel group whilst only one patient was from the Merocel group. This seems to support the notion that Merocel provides superior haemostatic durability, although this should be interpreted with discretion due to small sample size.

In our study, we highlight two clinically notable events which illustrated Merocel’s utility in managing post-operative bleeding and septal haematoma. One patient in the Surgicel group developed immediate post-operative epistaxis, requiring Merocel re-packing for control but was subsequently excluded from the study due to incomplete follow-up data. Another Surgicel patient presented with a septal haematoma at first outpatient visit, requiring drainage and temporary Merocel packing to prevent recollection of the haematoma. These cases highlight Merocel’s superiority in circumstances requiring mechanical tamponade, a property not offered by Surgicel due its mechanical properties. We postulate that a main contributor to post-operative complications experienced by these two patients may be attributed to their active smoking status – a known risk factor for increased post-operative bleeding.^12^

In addition to clinical outcomes, it is worthwhile to note both Merocel and Surgicel are priced comparably in our institution, with the cost of Merocel at approximately SGD 8.50 (or SGD 17 for 2) and Surgicel costing around SGD 18.54 per sheet used. As such, clinical decision-making can be more heavily guided by individual patient needs and surgical priorities.

Overall, based on the findings from our study, we propose that Merocel may be preferred for patients at higher risk of post-operative bleeding, while Surgicel may be more suitable for patients prioritising reduced nasal obstruction and discomfort in the immediate post-operative period.

### Limitations

Several limitations should be considered in the interpretation of our findings. First, the inclusion of patients undergoing concurrent septoplasty may limit generalisability to cases of inferior turbinoplasty without septoplasty. The presence of septal splints and septal incisions may have contributed to increased discomfort, pain and nasal obstruction during follow-up. However, although subgroup analysis comparing patients who underwent inferior turbinoplasty alone versus those who had concurrent septoplasty was considered, the sample size within each subgroup was insufficient to yield meaningful statistical comparisons. While influence of septoplasty-related factors cannot entirely be excluded, combined septoplasty and turbinate surgery reflects real-world clinical practice, as nasal obstruction in this population frequently involves both structural components. Restricting to inclusion of inferior turbinoplasty without septoplasty only would have compromised sample size and reduced external validity.

Secondly, procedures were performed by multiple surgeons, potentially introducing variability related to operator technique and experience. To mitigate this, surgical steps were standardised through pre-study training videos, and strict adherence was reinforced throughout the study period.

Additionally, the study relied on subjective outcome measures, including discomfort, pain and nasal obstruction, which may be influenced by individual perception. We aimed to enhance reliability and comparability by adopting validated scoring systems (e.g. VAS, TNSS nasal congestion subset).

Lastly, the short-term follow-up duration (up to POD7) limits our ability to evaluate longer-term complications such as delayed epistaxis beyond first post-operative week or synechiae formation. A 7-day follow-up period was selected to capture the immediate post-operative healing phase, during which most symptoms and packing-related outcomes manifest, while ensuring uniformity in follow-up and minimising attrition.

Further studies involving larger, multi-center trials with longer follow-up periods to further validate our findings and clarify the optimal indications for each type of nasal packing material.

## Conclusion

In summary, this study demonstrates that Merocel nasal packing is associated with increased nasal obstruction in the immediate POD 1 period. However, it confers superior prolonged haemostatic efficacy at the first outpatient follow-up when compared to Surgicel. Notably, despite the higher obstruction scores observed with Merocel, there were no significant differences in patient-reported discomfort or pain between the two groups. These findings suggest that both Merocel and Surgicel are clinically effective options for post-operative nasal packing following inferior turbinoplasty (with or without septoplasty), and the selection of packing material may be guided by patient-specific considerations and surgeon preference.

## Table 1 Long description

The table compares baseline demographics and clinical characteristics for two nasal packing methods: Merocel with 30 participants and Surgicel with 28 participants. Mean age was similar between groups, about 29 to 30 years, with comparable variability. Both groups were predominantly male, with roughly three quarters male and about one quarter female. Ethnicity differed somewhat: Chinese participants were more common in the Surgicel group, while Malay participants appeared only in the Merocel group; Indian and other ethnicities were present in both groups at smaller proportions. Most participants were non-smokers in both groups, but light smoking under five sticks per day occurred only in the Surgicel group; heavier smoking categories were uncommon overall. Septoplasty was performed for most participants in both groups, slightly more often in the Surgicel group. These figures describe group composition and do not indicate whether any differences are statistically meaningful.

Navigate back to Table 1.

## Table 2 Long description

The table compares Merocel (30 people) and Surgicel (28 people) on post-operative day 1 and post-operative days 5 to 7 for bleeding, discomfort, pain, and nasal obstruction, reporting mean with standard deviation, ranges, and adjusted p-values. On day 1, bleeding was similar (Merocel mean 1.00, Surgicel 1.18; adjusted p 0.146), as were discomfort (4.3 vs 3.54; adjusted p 0.249) and pain (about 1.3 vs about 1.1; adjusted p 0.818). Day 1 obstruction was higher in the Surgicel group (1.57) than the Merocel group (1.10), and this was the only statistically significant difference reported (adjusted p 0.008). On days 5 to 7, bleeding was higher with Surgicel (1.18) than Merocel (0.77), with a statistically significant adjusted p-value (0.044). For days 5 to 7, discomfort (2.63 vs 3.57; adjusted p 0.141), pain (1.80 vs 2.32; adjusted p 0.354), and obstruction (1.20 vs 1.32; adjusted p 0.548) did not show statistically significant differences. Ranges indicate overlapping score distributions between groups at both time points, so mean differences should be interpreted alongside variability and the nonparametric testing approach.

Navigate back to Table 2.
